# Post-dural puncture headache accompanied by obvious nasal congestion: A case report

**DOI:** 10.1097/MD.0000000000034078

**Published:** 2023-06-16

**Authors:** Ming Yan, Qiao Wang, Yufeng Zhang, Yue Sun, Jian Sun

**Affiliations:** a Department of Anesthesiology, The Huai’an Maternity and Child Clinical College of Xuzhou Medical University (Huai’ an maternal and child health care center), Huaian, China.

**Keywords:** case report, Epidural anesthesia, nasal congestion, Post-dural puncture headache

## Abstract

Post-dural puncture headache (PDPH) is the most common complication of intraspinal nerve block in gravida. PDPH may be accompanied by neck stiffness, tinnitus, hearing loss, photophobia or nausea.

**Patient concerns:**

A 33-year-old woman whose dura was accidentally punctured during labor analgesia complained of severe headache, dizziness, and nasal congestion; these symptoms aggravated when she looked up, and her sense of smell was normal 8 hours after catheter removal.

**Diagnoses:**

Based on the patient’s complaints and clinical appearance, diagnosis of PDPH was considered.

**Interventions:**

Nasal congestion disappeared with headache and dizziness after epidural injections of saline. The puerpera received saline injections 4 times; after treatment, she was discharged from the hospital when the symptoms did not limit her daily movement.

**Outcomes:**

The symptoms disappeared completely on the seventh day of telephone follow-up visit. The mechanism of her nasal obstruction is not very clear.

**Conclusion:**

We believe it is caused by the pulling of the intracranial nerve as the brain tissue sinks and shifts due to the decrease in intracranial pressure.

## 1. Introduction

Labour epidural analgesia can be complicated by accidental dural puncture which often leads to post-dural puncture headache (PDPH).^[1]^ PDPH is defined as any headache after lumbar puncture, which worsens within 15 minutes of sitting or standing and relieves within 15 minutes of lying down.^[2]^ PDPH hinders women ability to perform daily activities, including caring for their baby. It also delays hospital discharge, and leads to hospital re-admission and to potentially legal liability.^[3]^ PDPH may be accompanied by neck stiffness, tinnitus, hearing loss, photophobia or nausea.^[4]^ But to our knowledge, there are no reports of PDPH accompanied by nasal congestion.

## 2. Case presentation

The puerpera, 33 years old, height 158cm, weight 63kg, was diagnosed G3P1 to be delivered at 40 + 6 weeks, with no abnormalities in her past and personal history, and no obvious abnormalities in the auxiliary examination results. She entered the home delivery room for delivery and labor analgesia was performed when visual analog score score was 7. After signing the personal informed consent form, she received 500 mL sodium lactate Ringer intravenous infusion, as well as the maternal and fetal monitoring (noninvasive maternal blood pressure, electrocardiogram and oxygen saturation monitoring, fetal heart rate and uterine systolic pressure). In the left lateral position, the neuraxial analgesia puncture was performed through a paramedian approach between L2 and L3. After the epidural catheter was inserted, it was found that the clear liquid was sucked out during the negative pressure suction test before the test dose was injected. It was suspected that the catheter was inserted into the subarachnoid space, so the epidural block was changed to continuous subarachnoid block for labor analgesia. Labor analgesia started at 20:10 on December 6, 2020, the analgesic effect was satisfactory and the process of labor was smooth. After the fetus was delivered at 22:30, the parturient was asked to lie flat without pillow and immediately given 2000 mL sodium lactate Ringer solution intravenously. The catheter was removed at 00:30 on December 7, 2020 and the puerpera was sent back to the ward.

At 08:30 on December 7, 2020, the woman complained of severe headache, dizziness and nasal congestion, these symptoms aggravated when she looked up, and her sense of smell was normal. We considered the PDPH occurred and explained the drug therapy and epidural filling treatment plan in detail to the puerpera. Due to breastfeeding concerns, the puerpera refused the drug treatment and received the epidural saline filling method for treatment.

At 10:30 on December 7, 2020, epidural catheterization was performed through the median approach near L3-L4 space. After indwelling epidural catheter, saline was slowly injected several times. After 5 mL was injected, the symptoms of dizziness and headache were relieved. After 15 mL was injected, the symptoms of headache, dizziness and nasal congestion disappeared when she was lying supine. After 20 mL was injected, no headache, dizziness and nasal congestion occurred when the parturient was instructed to look up. At 17:00 on December 7, 2020, she complained that she did not get out of bed and sat up to eat twice for about 30 minutes during the day, and now she had symptoms of headache and dizziness when looking up. So the epidural space was filled with 20 mL normal saline again. At 08:30 on December 8, 2020, the puerpera complained that the symptoms were significantly alleviated compared with the previous day, but the symptoms were still severe when she raised her head. So we injected 20 mL saline into her epidural space again. At 08:30 on December 9, 2020, the puerpera said the symptoms were not obvious when raising head, but her movement was limited, so she was injected with 20 mL normal saline again. At 08:30 on December 10, 2020, the puerpera said she could get out of bed the previous day and her activities were not restricted, so she was discharged from the hospital. At 08:30 on December 13, 2020, the puerpera said the symptoms disappeared completely by telephone.

## 3. Discussion and conclusions

The puerpera in the case we reported presented with the adverse reaction of headache, dizziness and nasal congestion due to the perforation of the dura mater. To our knowledge, there was no previous description of the concomitant symptom of nasal congestion in PDPH. About 8 hours after the removal of the catheter into the subarachnoid space, she developed obvious symptoms of headache, dizziness and nasal obstruction, which became worse with the rise of her head. After the epidural saline filling treatment, the symptoms of nasal obstruction disappeared with the disappearance of headache and dizziness. In this process, the maternal sense of smell was normal.

During the operation, we did not draw back the cerebrospinal fluid when the epidural needle puncture was completed, but we found that the clear fluid was drawn back after the catheterization operation. We speculated that the dura mater had been punctured when the puncture needle was fixed, but a valve was formed. The valve blocked the tip of the needle when it was drawn back, so the cerebrospinal fluid was not pumped back. When the epidural catheter was placed, the valve was pushed away and tear might occur, and the catheter entered the subarachnoid space to form a large hole. So the woman symptoms appeared very early, showing unbearable symptoms only 8 hours after the catheter was removed.

At present, the exact mechanism of PDPH is not clear, which may be related to the following factors. First, because the cranial volume is fixed, the decrease of intracranial pressure after the outflow of cerebrospinal fluid leads to compensatory dilation of intracranial vessels and increase of blood flow, leading to the occurrence of PDPH.^[5]^ Second, reduced cerebrospinal fluid volume may directly activate adenosine receptors, leading to cerebral vasodilation and pain-sensitive brain structure stretching, resulting in PDPH.^[6]^ Third, the decrease of intracranial pressure will cause the brain tissue to sink and shift, which will stretch the intracranial nerves, meninges and blood vessels, resulting in the occurrence of PDPH.^[7]^

In this case, the symptoms appeared early, headache and dizziness were accompanied by obvious nasal congestion, while her sense of smell was normal, so it was speculated that this was caused by the traction of the parasympathetic nerve innervating the nasal cavity. The parasympathetic nerves in the nasal cavity come from the pterygoid canal nerve and the anterior ethmoidal nerve.^[8]^ The parasympathetic nerve of the pterygoid canal nerve comes from the great petrosal nerve, a branch of the facial nerve, and the anterior ethmoidal nerve originates from the ophthalmic branch of the trigeminal nerve. Parasympathetic nerve excitation can cause the nasal mucosa edema, vasodilation and increased secretion of glands in the nasal cavity, resulting in nasal congestion symptoms. However, the pregnant woman did not show symptoms of neck stiffness, tinnitus, hearing loss, photophobia or nausea, nor other symptoms of facial nerve or trigeminal nerve tension, only headache, dizziness and nasal congestion, which may be related to personal nerve variation.

Conservative treatment of PDPH includes bed rest, adequate hydration, use of NASIDs, narcotic analgesics or caffeine, etc.^[9]^ It has also been reported that there is no evidence that routine bed rest after dural puncture is beneficial for preventing the occurrence of PDPH.^[10]^ we took bed rest and adequate hydration to prevent the occurrence of PDPH immediately after the delivery of the fetus, but unfortunately PDPH still occurred very soon. Epidural blood patch is the gold standard for the treatment of PDPH.^[11]^ Complications of epidural blood patch include, but are not limited to, facial paralysis, back pain, permanent spastic paralysis, infection, cauda equina syndrome and meningitis.^[12]^ Less invasive treatments include epidural filling (saline or dextran 40 mg solution), sphenopalatine ganglion block, greater occipital nerve block and so on.^[13]^ After we introduced these therapies to the parturient, epidural saline therapy was requested for breast-feeding reasons. We gave 20 mL epidural filling with saline for a total of 4 times. The effect was very significant after each saline filling, but the duration of the effect is shorter than that of EPB therapy, and it needs repeated use to achieve satisfactory effects, which was consistent with some previous studies.^[14]^

The patient with PPDH in our report was accompanied by obvious nasal obstruction, and the symptoms of headache, dizziness and nasal obstruction disappeared immediately after normal saline filling treatment. The mechanism of her nasal obstruction is not very clear. We believe it is caused by the pulling of the intracranial nerve as the brain tissue sinks and shifts due to the decrease in intracranial pressure.

## Author contributions

**Investigation:** Ming Yan, Yufeng Zhang, Yue Sun, Jian Sun.

**Writing – original draft:** Ming Yan, Qiao Wang.

**Writing – review & editing:** Jian Sun.
